# Increased Cardiovascular Events and Subclinical Atherosclerosis in Rheumatoid Arthritis Patients: 1 Year Prospective Single Centre Study

**DOI:** 10.1371/journal.pone.0170108

**Published:** 2017-01-19

**Authors:** Piero Ruscitti, Paola Cipriani, Francesco Masedu, Silvio Romano, Onorina Berardicurti, Vasiliki Liakouli, Francesco Carubbi, Paola Di Benedetto, Saverio Alvaro, Maria Penco, Marco Valenti, Roberto Giacomelli

**Affiliations:** 1 Division of Rheumatology, Department of Biotechnological and Applied Clinical Science, University of L'Aquila, L'Aquila, Italy; 2 Division of Medical Statistic Unit, Department of Biotechnological and Applied Clinical Science, University of L'Aquila, L'Aquila, Italy; 3 Division of Cardiology, Department of Internal Medicine and Public Health, University of L'Aquila, L'Aquila, Italy; Beijing Key Laboratory of Diabetes Prevention and Research, CHINA

## Abstract

**Objectives:**

Several studies showed the close relationship between Rheumatoid Arthritis (RA) and cerebro-cardiovascular events (CVEs) and subclinical atherosclerosis. In this study, we investigated the occurrence of CVEs and subclinical atherosclerosis during the course of RA and we evaluated the possible role of both traditional cardiovascular (CV) and disease related risk factors to predict the occurrence of new CVEs and the onset of subclinical atherosclerosis.

**Methods:**

We designed a single centre, bias-adjusted, prospective, observational study to investigate, in a homogeneous subset of RA patients, the occurrence of new onset of CVEs and subclinical atherosclerosis. Statistical analyses were performed to evaluate the role of traditional CV and disease-related risk factors to predict the occurrence of new CVEs and subclinical atherosclerosis.

**Results:**

We enrolled 347 RA patients prospectively followed for 12 months. An increased percentage of patients experienced CVEs, developed subclinical atherosclerosis and was affected by systemic arterial hypertension (SAH), type 2 diabetes mellitus and metabolic syndrome (MS), at the end of follow up. Our analysis showed that the insurgence of both SAH and MS, during the follow up, the older age, the CVE familiarity and the lack of clinical response, were associated with a significantly increased risk to experience CVEs and to develop subclinical atherosclerosis.

**Conclusions:**

Our study quantifies the increased expected risk for CVEs in a cohort of RA patients prospectively followed for 1 year. The occurrence of both new CVEs and subclinical atherosclerosis in RA patients may be explained by inflammatory burden as well as traditional CV risk factors.

## Introduction

Rheumatoid arthritis (RA) is a chronic inflammatory autoimmune disease characterized by progressive joint destruction, associated with extra-articular manifestations, affecting different internal organs [1]. Interestingly, these patients show an increased risk of mortality when compared to general population and recent evidence clearly confirmed that this risk is largely due to cerebro-cardiovascular events (CVEs) [2,3]. In addition, several studies showed the close relationship between RA and specific cardiovascular (CV) events, including myocardial infarction (MI), cerebrovascular accident (CVA) and congestive heart failure (CHF) [4,5].

It is now well-known that increased subclinical atherosclerosis, mainly carotid artery plaques, may be observed in RA patients, which may be easily recognized by ultrasound, thus identifying those patients with higher CVEs risk [6]. In addition, multiple lines of evidence reported that CV risk factors are probably underestimated in RA patients [7], although the international recommendations clearly state about the assessment of this specific risk [8].

The evidence of traditional CV risk factors and subclinical atherosclerosis does not fully explain the increased incidence of CVEs in these patients; suggesting that the CV risk may be independently associated with RA and in fact, this risk has been shown to be associated with additional features specific of RA, such as the systemic inflammatory process, disease duration and therapeutic strategies [2,3,9,10].

It must be pointed out, that available data in this field derived from studies, generally retrospective or cross sectional or alternatively from medical records and registers [4,5,6,11]. It is well-known that these kind of studies may be affected by different biases, such as selection, reporting and recall biases, thus weakening the strength of the messages. Thus to better focus the occurrence of CVEs and subclinical atherosclerosis during the course of RA rheumatoid disease and to evaluate the related risk factors, we designed a single centre, bias-adjusted, prospective study in order to investigate the occurrence of new onset of CVEs and subclinical atherosclerosis, during 1 year of follow-up in our RA patients. Furthermore, we evaluated the role of both traditional CV and disease related risk factors to predict the occurrence of new CVEs and the onset of subclinical atherosclerosis.

## Patients and Methods

### Study design, inclusion criteria and data collection

In this prospective, single centre, bias-adjusted, observational study, we consecutively enrolled 347 RA patients, fulfilling 2010 ACR/EULAR and/or 1987 ACR diagnostic criteria [12,13], followed for 12 months, in the period ranging between 1/1/2010 and 31/12/2014. The patients were evaluated for traditional CV risk factors, history of CVEs or subclinical atherosclerosis, at the first visit (Time 0) and after 12 months (month 12), in order to identify the occurrence of any new CVE or the onset of subclinical atherosclerosis. CVEs were defined as occurrence of MI and/or CHF and/or CVA. Subclinical atherosclerosis was defined as the presence of carotid plaque at the ultrasound technique. Smoking habit, body mass index (BMI), familiarity for CVEs, the evidence of Systemic Arterial Hypertension (SAH), Type 2 diabetes (T2D), Metabolic syndrome (MS) as well as serum levels of total cholesterol, tryglycerides and glycemia were recorded at the Time 0 and after 12 months of follow-up. Patients were evaluated every 3 months, to record disease activity by Disease Activity Score in 28 joints (DAS28) and simplified disease activity index (SDAI), and the clinical response, according the EULAR improvement criteria [14].

The patients were divided, at Time 0, in 5 groups, according with their therapeutic regimen: i. single Synthetic Disease-Modifying Anti-Rheumatic Drug (SDMARD) +glucocorticoids (GCs); ii. combination of 2 or more SDMARDs; iii. SDMARD(s)+GCs+Biologic; iv. SDMARD(s)+Biologic; v. Other therapeutic regimen.

In this study, we strictly followed the international and national recommendations for the follow up and treatment of RA patients. The local ethics committee (Azienda Sanitaria Locale 1 Avezzano/Sulmona/L’Aquila, L’Aquila, Italy) approved this study. It has been performed according to the Good Clinical Practice guidelines, and written informed consent was obtained from all patients, according to the Declaration of Helsinki.

### Carotid ultrasound method

Carotid ultrasound was performed at the Time 0 and after 12 months of follow-up. Carotid ultrasound Arterial atherosclerotic plaques in the extracranial carotid tree were identified using the commercially available scanner, Mylab 70 Esaote (Genoa, Italy) equipped with a 7 to 12 MHz linear transducer and the automated software-guided technique radio frequency-Quality Intima Media Thickness in real-time (QIMT, Esaote, Maastricht, The Netherlands) was used. Carotid artery plaque was identified as recommended in the Mannheim consensus, that is when a focal structure that encroaches into the arterial lumen of at least 0.5 mm or 50% of the surrounding intima-media thickness (IMT) value or demonstrates a thickness of >1.5 mm as measured from the media-adventitia interface of the intima-lumen interface, is present [15].

### Statistical analysis

The statistical analysis dealt with a longitudinal design which focused the response pattern of the disease activity. McNemar test has been employed to test changes in comorbidities of the patients between the onset and the end of observation. Our analysis was aimed to assess the ORs, for the occurrence of both CVEs and subclinical atherosclerosis, identifying the covariates in the 2 main areas:

1. traditional CV risk factors: gender, age, familiarity, BMI, serum levels of total cholesterol, triglycerides and glycemia after 12 months of observation, the presence of MS, T2D and SAH after 12 months of observation;
2. RA-related risk factors: the duration of the disease, the clinical response, after 12 months of observation, according to the EULAR criteria.

We modelled 2 statistical analyses, adjusted for gender and age, by performing a logistic regression, in order to evaluate, in the first analysis, the predictive role of each identified covariate on CVEs occurrence, after 12 months of follow-up, in those patients not affected by CVE, and the second analysis in order to evaluate the predictive role of the same set of covariates on the occurrence of subclinical atherosclerosis. The models accounted for the interaction between age and familiarity. Models fitting has been assessed carrying out a log-likelihood ratio test Sidak adjusted for multiple tests with type I error set at 0.05. The analysis has been performed using STATA software (Version 14).

## Results

### Baseline characteristics of population

Three hundred forty-seven consecutive RA patients, fulfilling 2010 ACR/EULAR classificative criteria were enrolled. Furthermore, a large percentage of enrolled patients also fulfilled the 1987 ACR Criteria (Table 1).

**Table 1:** Baseline clinical characteristics.

Clinical features	Female	Male
Patients	295 (78.67%)	52 (21.33%)
Age, mean ± SD (Years)	60.95 ± 12.88	61.22 ± 16.39
ACR classificative criteria 1987	64.70%	73.10%
ACR/EULAR classificative criteria 2010	99.70%	98.1%
Disease duration (mean ± SD)	11.75 ± 7.01	11.01 ± 7.69
*< 1 years*	3.10%	5.80%
*between 1 and 5 years*	25.40%	25.00%
*between 5 and 10 years*	22.40%	30.82%
*≥ 10 years*	49.20%	38.53%
RF and/or ACPA	70.50%	71.22%
WBC (10^3^/mL) (mean ± SD)	5.61 ± 1.28	5.52 ± 1.36
RBC (10^3^/mL) (mean ± SD)	4.87 ± 0.54	4.85 ± 0.68
PLT (10^3^/mL) (mean ± SD)	238.28 ± 28.41	251.26 ± 31.01
Radiologic damage	24.09%	15.41%
Joint surgery	6.40%	7.80%
Extra-articular disease	14.6%	19.20%
*Pulmonary fibrosis*	4.43%	4.50%
*Sjogren’s Syndrome*	2.01%	2.02%
*Rheumatoid Vasculitis*	0.44%	1.00%
Disease activity_DAS28_Time 0 (mean ± SD)	4.09 ± 1.01	4.69 ± 1.04
*Remission*	0.71%	2.00&
*Low disease activity*	1.39%	0%
*Moderate disease activity*	58.60%	58.80%
*High disease activity*	6.80%	39.20%
SDAI_ Time 0 (mean ± SD)	24.21 ± 7.57	25.35 ± 8.87
HAQ-DI_ Time 0 (mean ± SD)	0.97 ± 0.64	0.68 ± 0.53
Smoke	27.11%	68.81%
BMI		
<18.49	4.69%	1.90%
>18.5 <24.99	60.71%	69.20%
>25 <29.99	27.82%	38.50%
>30	6.78%	0%
Familiarity_CVEs	34.50%	30.40%
Systemic Arterial Hypertension	43.09%	38.50%
Systolic blood pressure (mmHg) (mean ± SD)	128.31 ± 21.05	119.21 ± 10.11
Dystolic blood pressure (mmHg) (mean ± SD)	81.35 ± 4.23	79.39 ± 08.51
Heart rate (Beat/min) (mean ± SD)	71.32 ± 9.12	74.71 ± 7.95
Type 2 diabetes	10.81%	13.50%
Metabolic syndrome	32.50%	11.50%
Cholesterol mg/dl	196.41 ± 55.25	179.90 ± 56.21
Cholesterol>200 mg/dl	47.45%	30.76%
Tryglycerides mg/dl	116.98 ± 44.59	109.28 ± 37.71
Glycemia mg/dl	93.08 ± 17.24	88.25 ± 14.04
Glycemia >110 mg/dl	14.57%	21.15%

Abbreviations: RF: rheumatoid factor; ACPA: Anti–citrullinated protein antibody; WBC: white blood cell count; RBC: red blood cells; HB: hemoglobin; PLT: platelet count; DAS28: Disease Activity Score in 28 joints; SDAI: simplified disease activity index; HAQ-DI: health assessment questionnaire disability Index; BMI: body mass index, CV: cardiovascular, CVEs: cerebro-cardiovascular events.

As observed in Table 1, the majority of these patients showed a long disease duration (≥ 10 years) and serum positivity for Rheumatoid Factor (RF) and/or anti–citrullinated protein antibody (ACPA). At the enrollment, 40.08% out of patients showed a radiologic damage, assessed by radiologic examination of wrists and hands. Sixteen percent out of our patients were affected by different extra-articular complications, mainly pulmonary fibrosis. The most common therapeutic strategies were SDMARD+GCs and SDMARD(s)+GCs+Biologic. Methotrexate (MTX) and TNF inhibitors were the most common SDMARD and biologic prescribed, respectively (Table 2). The mean dosage of prednisone in these 2 strategies was 7.5±2.5 mg/daily.

**Table 2:** Therapeutic strategies of the enrolled patients at the first visit.

Therapeutic scheme	
*SDMARD + GCs*	39.77%
*SDMARDs combination*	10.95%
*SDMARD(s) + GCs+ Biologic*	37.46%
*SDMARD(s) + Biologic*	7.49%
*Other*	4.32%
SDMARD	95.68%
*MTX*	78.38%
*Other_SDMARDs*	17.30%
Biologics	49.27%
*TNF inhibitor*	36.19%
*Other_biologics*	13.08%

Abbreviations: SDMARD: synthetic disease-modifying anti-rheumatic drug; GCs: glucocorticoids; MTX: methotrexate; TNF: tumor necrosis factor.

### Effectiveness and disease activity evaluation

**Fig 1:**
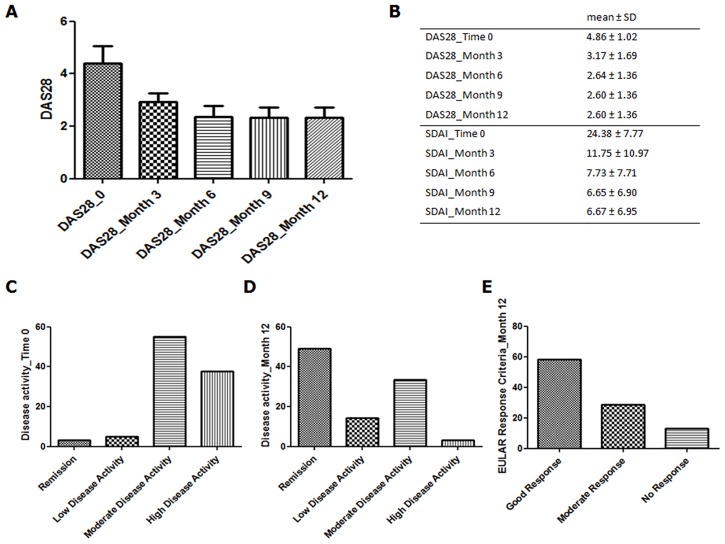
Effectiveness and disease activity evaluation in the enrolled patients. This Figure shows: A) and B) the progressive reduction of both DAS28 and SDAI values; C) the disease activity at the first observation and after 12 months of follow up, respectively; E) the EULAR response criteria after 12 months of follow up.

**Fig 2:**
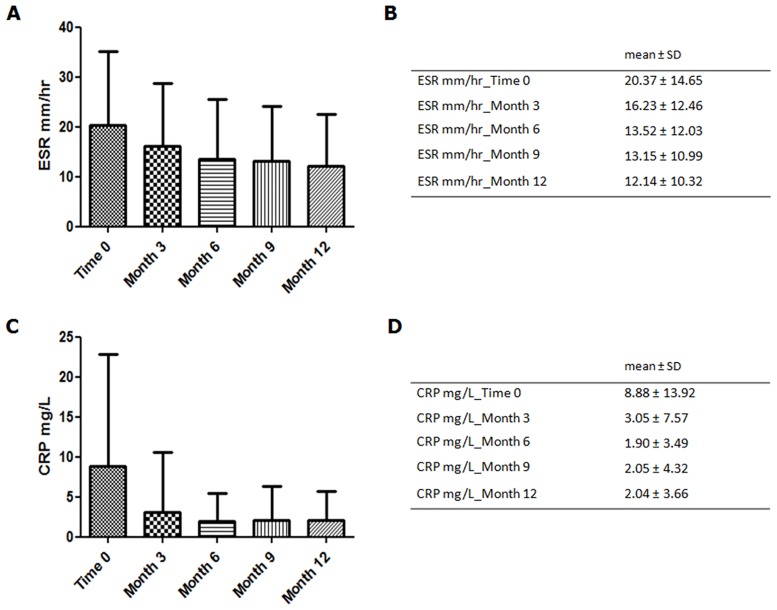
Evaluation of pro-inflammatory markers. This Figure shows: A) and B) the progressive reduction of the serum mean levels of ESR; C) and D) the progressive reduction of the serum mean levels of CRP, during the follow up.

The enrolled patients were evaluated, every 3 months, to record the disease activity. During the follow up, we observed a progressive reduction of both DAS28 and SDAI values (Fig 1). At month 12, 49% out of patients reached DAS28 remission (<2.6) and 14.4% reached low disease activity. Totally, 58.22% out of the enrolled patients reached a good clinical response, according with EULAR criteria, at the end of follow up. Furthermore, a progressive reduction of both ESR and CRP mean levels was observed, as shown in Fig 2.

### Traditional CV risk factors: Comparison between time 0 and month 12

**Fig 3:**
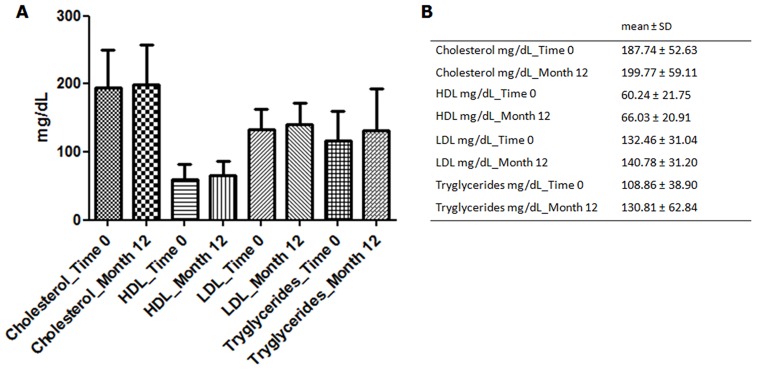
Comparison of lipid profile between time 0 and 12 month. This Figure shows: A) and B) the mean serum levels of total cholesterol, HDL, LDL triglycerides at the first observation and after 12 months of follow up, respectively.

As far as the traditional CV risk factors are concerned, 42.36% out of enrolled patients were affected by SAH, 34.50% showed a familiarity for CVEs, 32% reported smoking habit, 29.39% were affected by MS and 11.24% by T2D. The majority of our patients showed a normal value of BMI (BMI>18.5 <24.99), at Time 0. At month 12, a new evaluation of risk factors and a comparison between time 0 and month 12 were performed. No significant difference was observed for both BMI values and smoking habit. Similarly, no difference was reported, analyzing the serum blood values (mean±SD) of total cholesterol (Time 0: 187.74±52.63 mg/dL vs month 12: 199.77±59.11 mg/dL) and triglycerides (Time 0: 108.86±38.90 mg/dL vs month 12 130.81±62.84 mg/dL). Fig 3 shows the lipid profile, of our patients, at Time 0 and at month 12. Although the mean values of glycaemia did not differ between Time 0 and month 12 (Time 0: 88.90±13.34 mg/dL vs month 12: 89.51±21.91 mg/dL, not significant), we observed a significant increase of patients with glycaemia levels ≥ 110 mg/dL at month 12 [Time 0: 46 patients, (13.54%) vs month 12: 64 patients (18.20%); p = 0.0003]. Finally, at month 12, we observed, in our cohort, a significantly increased number of patients affected by SAH [Time 0: 146 patients (42.36%) vs month 12: 193 patients (55.62%); p<0.00001], MS [Time 0: 101 patients (29.39%) vs month 12: 153 (44.09%); p<0.00001] and T2D [Time 0: 39 patients (42.36%) vs month 12: 61 patients (42.36%); p<0.00001], when compared with Time 0 (Fig 2).

### New CVEs onset and related factors

**Fig 4:**
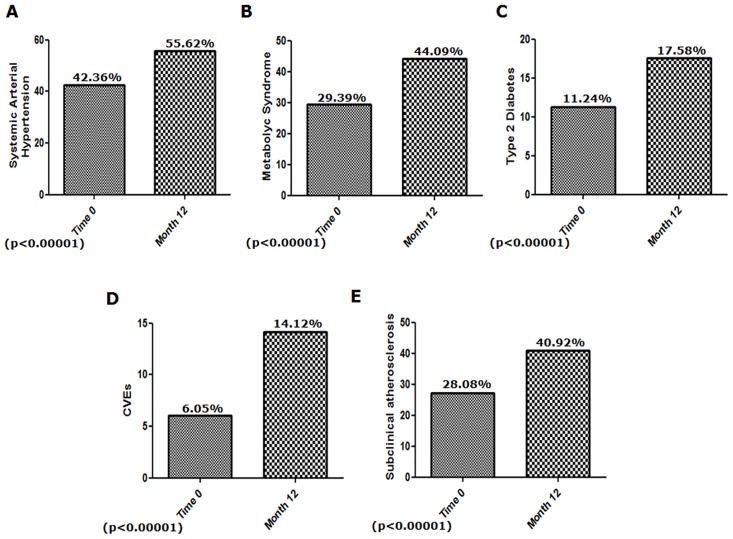
Traditional CV risk factors, CVEs and subclinical atherosclerosis, comparison between time 0 and 12 month. This Figure shows: A), B) and C) the increased percentages of patients affected by SAH, MS and T2D (p<0.00001 for each comparison), after 12 months of follow up. D) and E) After 12 months, we observed an increased percentage of patients experienced new CVEs and developed subclinical atherosclerosis, respectively (p<0.00001 for each comparison).

Six percent of patients had a positive history for CVEs at Time 0. After 12 months, as shown in Fig 4, we observed a 2-fold increase of this percentage (14.12%), and this difference was statistically significant when compared with the percentage at Time 0 (p<0.00001). Furthermore, 34 patients out of 326 patients, without history of CVEs, experienced, at least, 1 new CVE during the follow up, specifically: 30 MI, 22 CHF and 3 CVAs. Table 3 shows the demographic features in the subset of patients with CVEs and subclinical atherosclerosis at Time 0 and after 12 months.

**Table 3:** Demographic features in the subset of patients with CVEs and subclinical atherosclerosis at the first observation and after 12 months of follow up.

**Disease activity and therapeutic strategies**	**CVEs_Time 0**	**CVEs_month 12**
Patients (%)	6.05%	14.12%
DAS28 (mean ± SD)	4.52 ± 0.70	3.71 ± 0.97
*Remission*	0	4.10%
*Low disease activity*	4.37%	32.70%
*Moderate disease activity*	80.85%	46.90%
*High disease activity*	14.78%	16.03%
No EULAR Response_12 month		22.39%
Moderate EULAR Response_12 month		51.01%
Good EULAR Response_12 month		26.50%
Therapeutic strategy		
*SDMARD(s) + GCs*	80.85%	46.90%
*SDMARDs combination*	0	12.21%
*SDMARD(s) + GCs+ Biologic*	14.78%	26.49%
*SDMARD(s) + Biologic*	4.37%	10.18%
*Other*	0	4.12%
**Disease activity and therapeutic strategies**	**Subclinical atherosclerosis_Time 0**	**Subclinical atherosclerosis_month 12**
Patients (%)	28.08%	40.92%
DAS28 (mean ± SD)	5.38 ± 0.93	3.34 ± 1.06
*Remission*	0	23.20%
*Low disease activity*	1.13%	18.30%
*Moderate disease activity*	42.57%	49.30%
*High disease activity*	56.40%	9.20%
No EULAR Response_12month		33.81%
Moderate EULAR Response_12 month		46.49%
Good EULAR Response_12 month		19.70%
Therapeutic strategy		
*SDMARD(s) + GCs*	53.20%	50.02%
*SDMARDs combination*	7.40%	4.90%
*SDMARD(s) + GCs+ Biologic*	28.70%	35.88%
*SDMARD(s) + Biologic*	8.47%	6.31%
*Other*	2.13%	2.79%

Abbreviations: CVEs: cerebro-cardiovascular events; SDMARD: synthetic disease-modifying anti-rheumatic drug; GCs: glucocorticoids.

Our analysis (Table 4) suggested that both age and CVEs familiarity were significantly associated with the development of new CVEs (p = 0.001, for both factors), and that the impact of CVEs familiarity increases with increasing age of the patients (p = 0.001). Furthermore, we showed that the insurgence of SAH, during the follow up, was associated with a significantly increased risk to experience CVEs (p = 0.04). At month 12, we observed that the lack of clinical response significantly correlated with an increased risk to develop new CVEs (no EULAR-DAS28 response p = 0.02; moderate EULAR-DAS28 response p = 0.03). In our study, smoking habit, BMI, total cholesterol values, glycaemia, disease duration and both MS and T2D insurgence were not associated with the risk to develop new CVEs.

**Table 4:** Predictive factors of new CVEs onset.

CVEs_ month 12	OR	SE	P	CI 95%
Gender	0.80	0.84	0.84	0.10–6.37
**Age#familiarity_No**	**1.16**	**0.05**	0.001*	**1.06–1.27**
**Age#familiarity_Yes**	**1.18**	**0.05**	0.001*	**1.07–1.29**
Smoke	0.99	0.77	0.99	0.21–4.60
BMI	1.42	0.71	0.48	0.53–3.79
Cholesterol_month 12	1.00	0.03	0.67	0.98–1.01
**Triglycerides_month 12**	**1.01**	**0.00**	0.02*	**1.00–1.02**
Glycemia_month 12	0.98	0.01	0.33	0.95–1.01
MS	0.43	0.36	0.319	0.08–2.24
T2D	0.36	0.30	0.22	0.07–1.85
**SAH**	**12.63**	**16.39**	0.04*	**0.99–160.62**
Disease duration	0.99	0.45	0.92	0.91–1.08
**No EULAR response_month 12**	**8.26**	**7.52**	0.02*	**1.38–49.20**
**Moderate EULAR response_month 12**	**5.34**	**4.13**	0.03*	**1.17–24.32**
Good response_month 12	1			

Abbreviations: CVEs cerebro-cardiovascular events; BMI: body mass index; SAH: Systemic Arterial Hypertension; MS: Metabolic syndrome; T2D: Type 2 diabetes.

*:statistically significant

### New subclinical atherosclerosis onset and related factors

Twenty eight percent of patients were positive for history of subclinical atherosclerosis, at Time 0. After 12 months, we observed an increase of this percentage (40.92%), and the difference with the percentage of patients with subclinical atherosclerosis at Time 0 was statistically significant (p<0.00001). Fifty patients, out of 254 patients without evidence of subclinical atherosclerosis, developed new carotid plaques during the follow up.

Our analysis (Table 5) showed that the insurgence of both SAH and MS, during the follow up, was associated with a significantly increased risk to develop subclinical atherosclerosis (p = 0.001, p = 0.04, respectively). At month 12, we observed that the lack of clinical response significantly correlated with an increased risk to develop new subclinical atherosclerosis (no EULAR-DAS28 response, p = 0.001). In our study, smoking habit, BMI, total cholesterol, triglycerides values, glycaemia, T2D and disease duration were not associated with risk to develop new subclinical atherosclerosis.

**Table 5:** Predictive factors of new subclinical atherosclerosis onset.

Subclinical atherosclerosis_ Month 12	OR	SE	P	CI 95%
Gender	1.18	0.66	0.76	0.39–3.58
Age#familiarity_0	1.03	0.02	0.19	0.98–1.07
Age#familiarity_1	1.02	0.02	0.24	0.98–1.07
Smoke	0.79	0.33	0.58	0.91–1.82
BMI	0.78	0.27	0.49	0.39–1.56
Cholesterol_month 12	0.99	0.00	0.96	0.99–1–00
Triglycerides_month 12	1.01	0.00	0.90	0.99–1.01
Glycemia_month 12	0.99	0.01	0.65	0.97–1.01
**MS**	**4.07**	**2.65**	0.001*	**1.55–14.23**
T2D	1.12	0.66	0.84	0.35–3.55
**SAH**	**2.69**	**1.33**	0.04*	**1.02–7.09**
Disease duration	0.96	0.32	0.31	0.90–1.03
**No EULAR response_month 12**	**6.74**	**3.86**	0.001*	**2.19–20.70**
Moderate EULAR response_month 12	2.35	1.17	0.08	0.88–6.27
Good response_month 12	1			

Abbreviations: BMI: body mass index; SAH: Systemic Arterial Hypertension; MS: Metabolic syndrome; T2D: Type 2 diabetes.

*:statistically significant

## Discussion

This study clearly shows that in our RA cohort, prospectively followed for 1 year, at month 12, the percentage of patients with CVEs and/or displaying subclinical atherosclerosis doubles, when compared with the same items at the beginning of the observation. Furthermore, we identified some traditional CV risk factors as well as RA-related factors which are involved in the development of CV complications.

In this context, it is well known that the inflammation may play a pivotal role in the atherosclerotic process, including endothelial dysfunction, plaque formation, which may evolve toward plaque destabilization and, finally, rupture [16]. Rheumatoid chronic inflammatory state is generally considered to increase CV risk, via different mechanisms, such as oxidative stress, endothelial dysfunction, pro-thrombotic state and pro-atherogenic metabolic effects [16,17]. Our bias-adjusted, single centre, prospective study, shows that the failure in fully control the inflammatory process increases from 4- to 8-fold the risk to develop both CVEs and subclinical atherosclerosis, thus suggesting the need of a good control of RA activity, to prevent the occurrence of CV complications [5,6]. Conflicting results may be available in literature concerning the association between disease duration and CV risk [9,18,19]. Differently from others reports, our study failed to recognize a positive association between disease duration and both CVEs and subclinical atherosclerosis. Our results might parallel published data suggesting in the last years, that the burden of the inflammatory process over time, more than RA duration strongly contribute in enhancing the CV risk [16,20,21]. Taken together, our data confirm the possible role of an active inflammatory process in increasing the risk of CVEs, in those RA patients with a persistent poorly controlled, active disease.

Due to the specifically design of our study, in which the treatments of RA patients were not randomized, we did not analyze any possible association between the treatments and the outcomes, thus avoiding the risk of a “confounding by indication” bias, a bias deriving when physicians decided to prescribe a more intensive treatment to those patients that, in their opinion, are affected by a more aggressive disease [22]. In this context, the lack of RCTs, specifically designed to evaluate the effect of different drugs in controlling the insurgence of CVEs, strongly limits the possibility to reach robust conclusions.

As far as traditional CV risk factors are concerned, in our study, we observed that after 12 month of follow up, a significant increase of patients were affected by SAH, T2D and MS. Recently, meta-analytic data, clearly confirmed that some traditional CV risk factors may contribute to CVEs development, in RA patients [7]. In fact, we observed that the development of SAH was strongly predictive of both CVEs and subclinical atherosclerosis, mirroring data reported in previous studies [23,24]. During RA, the increased expression of TNF-α, IL-1β, IL-6, adhesion molecules, angiotensin II type 1 receptor, and endothelin, together with a lower expression of NO, might contribute to SAH via endothelial dysfunction [25]. At present, available SDMARDs did not show any positive effect in controlling systemic blood pressure; on the contrary, NSAIDs and may rise the systemic blood pressure [23,26]. Furthermore, it must be pointed out that, as recently reported, SAH may not be optimally identified, in RA patients [27], thus contributing to the increase of CVEs risk.

To date, multiple lines of evidence confirmed a significant association between RA and MS [28,29]. Our prospective study shows a significantly increased percentage of RA patients displaying MS, at month 12,when compared with the MS percentage at Time 0. In this clinical setting, we observed that MS is associated with a 4-fold increased risk to develop subclinical atherosclerosis.

Despite of the well-known role of traditional CV risk factors, in inducing CVEs and subclinical atherosclerosis in general population, our analysis failed to show any possible association among some of these factors and the development of CVEs, in RA. As far as T2D is concerned, although the percentage of RA patients that developed T2D, at month 12, was increased, we did not find any association between the development of T2D and the insurgence of new CVEs and subclinical atherosclerosis. In our opinion, this lack of predictivity may be related to the short time of follow up, which could be not sufficient to evaluate the deleterious effects of T2D on CVEs. In addition, a large percentage of our patients were treated with GCs, and it is well-known the association between this drug and the insurgence of T2D [30]. On these bases, current EULAR recommendations suggest to limit the use of GCs to the lowest dose and for the shortest possible duration [8,31,32].

Although the increased prevalence of smoking habit in RA patients is well established [1], its impact on CVEs and subclinical atherosclerosis, during RA, have still not been identified [18,33]. A weaker association between smoking and CVEs, in RA patients, has been previously shown [18,34] and, this “smoking paradox” might be related with a well-known “index event” bias, a frequent bias of observational and epidemiological studies in which causal factors appear not to apply to disease complications [35].

We further evaluated the impact of both body weight and obesity, on CVEs and subclinical atherosclerosis via the assessment of BMI. In our RA cohort, we did not find any significant association between BMI and outcomes. BMI is a validated index assessing obesity at the whole-body level; however, during RA, in which sarcopenia alters the body composition, BMI may not be considered a valid predictor of CVEs and subclinical atherosclerosis as in normal population [36,37]. Although other anthropometric measures of central adiposity, such as waist circumference and waist to hip ratio, have been proposed as alternatives to BMI [36,37], further studies are needed to identify the optimal method to assess the potential role of obesity as a predictor for CV comorbidities.

As far as the role of dyslipidaemia in RA is concerned, we did not observe any association between the serum values of total cholesterol and both CVEs and subclinical atherosclerosis. In fact, the quantitative analyses may not identify the real impact of serum of total cholesterol on CVEs development during RA. As far as the potential pro-atherogenic role of lipoproteins, during RA, is concerned, it has been suggested that despite the increased CV risk observed during this disease, the inflammatory process induces a decrease of serum total cholesterol, via the activity of pro-inflammatory cytokines (RA lipid paradox) [38]. On the other hand, the systemic inflammation modulates specific qualitative changes in lipoproteins, mainly affecting the HDL fraction, which loses its anti-inflammatory activity and its skill to reverse cholesterol transport function [39,40]. Further studies are needed to fully clarify the role of lipoproteins in inducing CVEs in RA patients [38].

## Conclusions

In conclusion, our prospective longitudinal observational study, overcomes some possible limitation, which are correlated with the specific design of clinical trials [41] and quantifies the increased expected risk for CVEs in a cohort of patients prospectively followed for 1 year. The occurrence of both new CVEs and subclinical atherosclerosis in RA patients may be explained by inflammatory burden as well as traditional CV risk factors. At present, we identified that the failure in suppressing the inflammatory process is the main RA-related variable, influencing the risk to develop new CVEs. As far as the traditional CV risk factors are concerned, we showed that SAH, MS, familiarity for CV diseases and older age are significantly associated with the risk to develop new CVEs. Furthermore, our results strongly suggest the need of optimal control of the disease activity to prevent the insurgence of new CVEs. Future longitudinal analyses, on larger cohorts of patients, with longer follow-up may reinforce these data, and suggest the better therapeutic strategies to prevent the occurrence of CVEs, which are, at present, the main cause of death in these patients.
